# Influence of Parental Monitoring, Sensation Seeking, Expected Social Benefits, and Refusal Efficacy on Tobacco and Alcohol Use in Chinese Adolescents

**DOI:** 10.1097/MD.0000000000002814

**Published:** 2016-03-18

**Authors:** Jincong Yu, Qingfeng Wu, Chengwu Yang, Kent E. Vrana, Li Zhou, Longyu Yang, Hui Zhang, Dong Yan, Jiang Li, Shiwei Teng, Jie Gong, Yaqiong Yan, Zengzhen Wang

**Affiliations:** From the Department of Epidemiology and Biostatistics (JY, QW, LY, HZ, DY, JL, ST, ZW), School of Public Health, Tongji Medical College, Huazhong University of Science and Technology, Wuhan, China; Division of Biostatistics (CY), Department of Public Health Sciences, and Office for Scholarship in Learning and Education Research (OSLER), College of Medicine, Pennsylvania State University, Hershey, PA; Department of Pharmacology (KEV), College of Medicine, Pennsylvania State University, Hershey, PA; Shenzhen Center for Disease Control and Prevention (LZ), Shenzhen, China; Chronic Disease Department (JG, YY), Wuhan Center for Disease Control and Prevention, Wuhan, China; Department of Preventive Medicine (QW), Gannan Medical University, Ganzhou, China; and Chongqing Health Information Center (JL), Chongqing, China.

## Abstract

The relationships between parental monitoring (PM), sensation seeking (SS), expected social benefits (ESB), refusal efficacy (RE), and tobacco and alcohol use (TAU) have been well documented among adolescents. However, the mechanisms by which these 4 determinants affect TAU remain unclear. Based on the Theory of Triadic Influence, this study aimed to explore how PM, SS, ESB, and RE simultaneously influenced TAU in Chinese adolescents.

From September 2013 to June 2014, we used multistage cluster sampling to select 6269 students from 179 classes of 7 vocational high schools in 3 cities of China. Each student completed a battery of 5 measures: PM, SS, ESB, RE, and TAU. Then, we used structural equation modeling techniques and mediation analyses to investigate the relationships among these 5 measures, with TAU as the final dependent variable.

Results demonstrated that the relationship between PM and TAU was fully mediated by ESB and RE (b = −0.18, *P* < 0.001), that SS influenced TAU directly (b = 0.10, *P* < 0.001) and indirectly through ESB and RE (b = 0.15, *P* < 0.001), and that ESB influenced TAU directly (b = 0.09, *P* < 0.001) and indirectly through RE (b = 0.28, *P* < 0.001).

These findings indicate that the link between PM and SS to TAU among Chinese adolescents can be explained by ESB and RE. These 4 precursory determinants can play an important role in TAU prevention among adolescents in China.

## Abstract 2

Supplemental Digital Content is available in the text

## INTRODUCTION

Tobacco and alcohol use (TAU) are prevalent among adolescents in China.^1,2^ Previous studies reported that the prevalence of lifetime versus current tobacco use in this population was 32.8% versus 9.0%^2^ and that the prevalence of lifetime versus current alcohol use was 51.1% versus 25.2%.^1^ TAU are related with numerous health and behavior problems such as cancer, cardiovascular disease, stroke,^3^ and illicit drug use.^4^ In view of the health consequences associated with TAU, it is imperative to take measures to control them, especially among adolescents. In order to do so, the first step is to find the factors that may influence TAU among adolescents.^5,6^

A series of theories have been proposed to explain the initiation and maintenance of substance use.^7^ One major contemporary theory is the Theory of Triadic Influence (TTI).^5,8,9^ The TTI organizes determinants of substance use in the personal, social, and environmental streams of influence. Meanwhile, in consideration of the influencing strength on substance use, the determinants within each stream are distinguished into ultimate, distal, and proximal levels. Ultimate determinates could influence substance use through distal and proximal determinates as mediators. Moreover, distal determinates could influence substance use through proximal determinates as mediators. These mediation effects occur within the same stream, as well as across streams. The TTI has widely served as a theoretical basis for determining the influential factors of substance use.^5^ However, there have been few studies to explore the determinants of substance use among Chinese adolescents from a perspective of this theory. The present study fills this knowledge gap and explores how parental monitoring (PM), sensation seeking (SS), expected social benefits (ESB), and refusal efficacy (RE) influence TAU based on the TTI.

PM and SS are ultimate determinants within the social and personal streams, respectively. ESB, a variable that depicts positive expectancies, is a distal determinant within the environmental stream. Meanwhile, RE is a proximal determinant within the personal stream. The relationships between these 4 determinants and substance use, including TAU, have been well documented among adolescents.^10–16^ Moreover, a substantial literature has focused on the mechanisms by which these determinants influence adolescent substance use.

Watkins et al^12^ reported that RE mediated the relationship between PM and adolescent alcohol use. However, the mediation effects of positive expectancies between PM and substance use remained unclear. Macaulay et al^17^ reported that ESB mediated the relationship between parental practices (PM as an indicator variable for this latent construct) and drug use. Another study demonstrated that low levels of PM could predict high levels of positive expectancies.^18^ Combined with direct associations of PM and positive expectancies on TAU,^11,16,18^ we speculated that ESB mediated the relationship between PM and TAU.

Furthermore, the mediation effects of positive expectancies between SS and TAU have been well established by Urban et al,^13,14^ whereas few studies explored the mediation effects of RE between SS and substance use. Fortunately, impulsivity, a personality trait closely interrelated with SS,^19,20^ has been reported that its relationships with alcohol and marijuana problems were mediated by RE.^21,22^ Hence, we speculated that a similar mediation mechanism existed between SS and TAU. In addition, the findings of Sun et al^23^ indicated that RE could mediate the relationship between positive alcohol expectancies and alcohol use among Chinese adolescents. Similar findings were reported among the samples of young adults, treatment-seeking substance abusers, and college students.^22,24,25^

**FIGURE 1:**
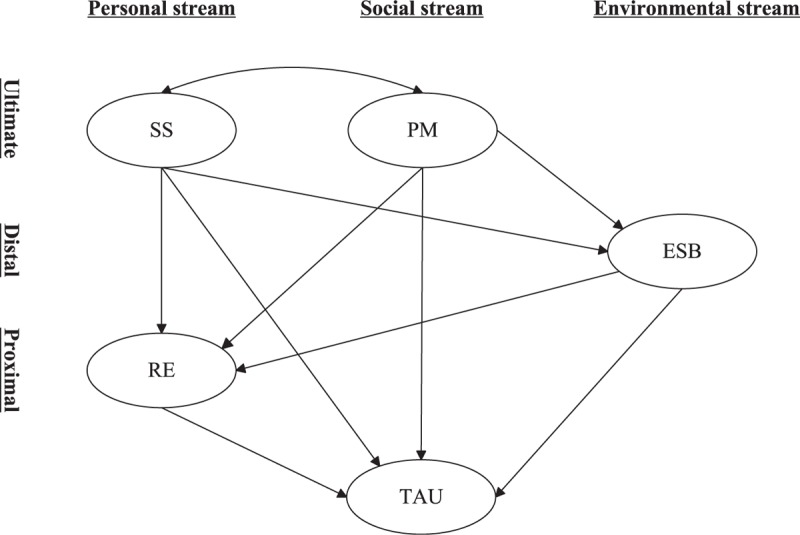
Proposed structural equation model consisted of PM, SS, ESB, RE, TAU among Chinese adolescents based on the TTI. Ovals represented latent variables and paths for indicators of latent variables were not shown for brevity. ESB = expected social benefits, PM = parental monitoring, RE = refusal efficacy, SS = sensation seeking, TAU = tobacco and alcohol use, TTI = Theory of Triadic Influence.

Taken together, despite the fact that some mechanisms how PM, SS, ESB, and RE influence TAU among adolescents have been reported, other mechanisms remain unclear and need to be further explored. The present study would provide the complementary evidence on this area. Based on the TTI and previous research, a structural equation model (SEM) consisting of PM, SS, ESB, RE, and TAU among Chinese adolescents was proposed in the present study (see Figure 1). Two exogenous variables (i.e., PM and SS) were allowed to covary freely. As shown in Figure 1, we set out to test 3 hypotheses: the relationship between PM and TAU was partially mediated by ESB and RE; the relationship between SS and TAU was partially mediated by ESB and RE; and RE partially mediated the relationship between ESB and TAU.

## METHODS

### Participants

**FIGURE 2:**
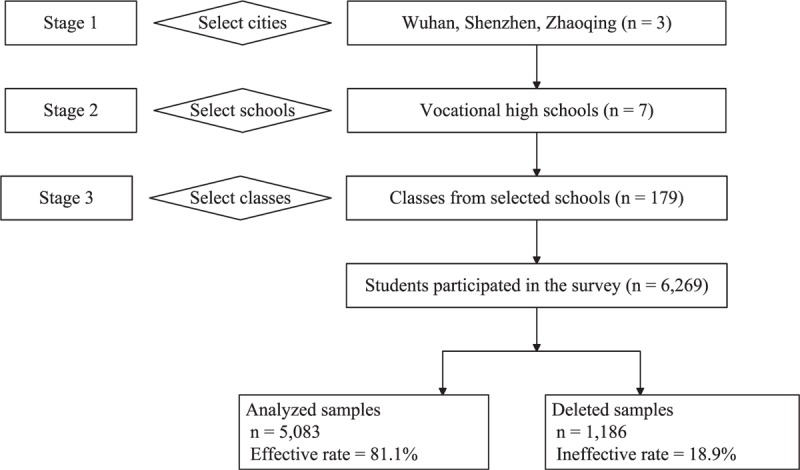
Flow chart for process of sampling in the present study.

Data were collected from September 2013 to June 2014 as part of an epidemiology survey of substance use disorders among Chinese vocational high school students. This population accounted for 44.5% of Chinese students in senior secondary education in 2013.^26^ In China, vocational high school provides a 3-year vocational/technical curriculum for students who usually have lower test scores than senior high school students. Most of them enter into the general workforce upon graduation. Multistage cluster sampling was adopted to select participants in the present study (see Figure 2). In stage 1, 3 cities (i.e., Wuhan, Shenzhen, and Zhaoqing) were purposively selected from Hubei and Guangdong provinces. Wuhan is the provincial capital city of Hubei. Shenzhen is the first Special Economic Zone of China located in Guangdong province. Zhaoqing is the first Vocational and Technical Education Training Base in Guangdong Province (established in 2005).^27^ In stage 2, vocational high schools were purposively selected from each city. Three schools were selected in Wuhan and 2 schools each were selected in Shenzhen and Zhaoqing (7 schools total). In stage 3, all or randomly selected classes were chosen from the 10th and 11th grade within each school. Students who agreed to participate in the survey within the selected classes were recruited. We did not include the 12th grade students because they were generally practicing (as vocational student interns) outside of the schools. Overall, a total of 6269 students in 179 classes (87 from the 10th grade and 92 from the 11th grade) participated in the survey. There were 1186 questionnaires (18.9%) deleted based on strategies of data quality control (specifics detailed below). So the number of the effective questionnaires was 5083, yielding an effective rate of 81.1%.

### Ethics Statement and Data Collection

At the design stage and before data collection from the field survey, we obtained Institutional Review Board approval for the conduct of this study from the Medical Ethics Committee (MEC) of Tongji Medical College, Huazhong University of Science and Technology. The MEC scrutinized the study design, especially the sections related to the field survey, so that all ethical norms including the Helsinki norms were met. In addition, at each of the 7 participating schools, we obtained agreement from the principals before we conducted the survey, given that all of the participating students are minor of age and that most of them are remote from their parents or guardians. Moreover, at the beginning of field survey, before distributing the paper-and-pencil questionnaires, the investigators took 5 minutes to clearly elucidate the purpose and content of the survey for participants, and the principles of confidentiality and voluntariness were emphasized to protect the participating students. The students were all informed that their responses were anonymous, and that none of their parents, teachers, or peers would be aware of their responses. Moreover, they could choose not to participate or to quit the survey at anytime without any questions asked. A total of 159 students refused to participate in the survey. In addition, the class teachers were asked to leave the classroom during administration of the survey. Data were collected during a single 40-minute classroom period by trained investigators following a standardized protocol. All data collection materials were in Chinese.

## MEASURES

Data were collected with a combination of modified questions from previously published survey instruments (as described below). For details on the questionnaire items, please refer to the Supplementary Table 1. The scale for *TAU* included the frequency and amount of TAU.^28^ The frequency of TAU was measured by 3 items rated on a 5-point scale from 1 (never) to 5 (daily). The amount of tobacco use per day was measured by a single item with options ranging from (1) “I don’t smoke” to (8) “more than 2 packs.” The amount of alcohol use per drinking occasion was measured by another single item with response options ranging from (1) “I don’t drink” to (6) more than 6 drinks.” These 2 items were scored on a 5-point scale corresponding to the scales for the frequency of TAU.

*PM* is a set of correlated parenting behaviors involving attention to and tracking of the child's whereabouts and activities.^29^ This variable was assessed with 5 items rated on a 4-point scale from 1 (strongly disagree) to 4 (strongly agree). The scale was originally developed as a family influential factor in the Communities That Care Youth Survey.^30^

*SS* is defined as the tendency to constantly seek novel, varying, and stimulating experiences and sensations, as well as the willingness to accept risk to obtain such arousal.^31^ This variable was measured by the Brief Sensation Seeking Scale for Chinese (BSSS-C).^32^ BSSS-C consisted of 4 subscales (i.e., experience seeking, boredom susceptibility, thrill and adventure seeking, and disinhibition). Each subscale was assessed by two 5-point Likert-type items ranging from 1 (strongly disagree) to 5 (strongly agree).

The scale for *ESB* was composed of eight 5-point Likert-type items ranging from 1 (strongly disagree) to 5 (strongly agree). This scale included 2 sets of similar items to assess social benefit expectancies for TAU (e.g., being cool or more grown-up), respectively.^33^

The *RE* scale included 2 items.^34^ Response options ranged from 1 (definitely would) to 5 (definitely would not). Both items were scored in reverse and high values indicated greater confidence to refuse the offers of tobacco or alcohol.

For the scales of ESB and PM, a standard forward-backward procedure was followed by several independent translators and minor changes were made to account for cultural differences. As shown in Table 2, Cronbach alphas (as a measure of internal consistency for each set of assessments) of all 5 measures were >0.70 and reached an adequate level of reliability. The demographic characteristics included age, sex, grade, residence, only child status, and family structure.

**TABLE 2:**
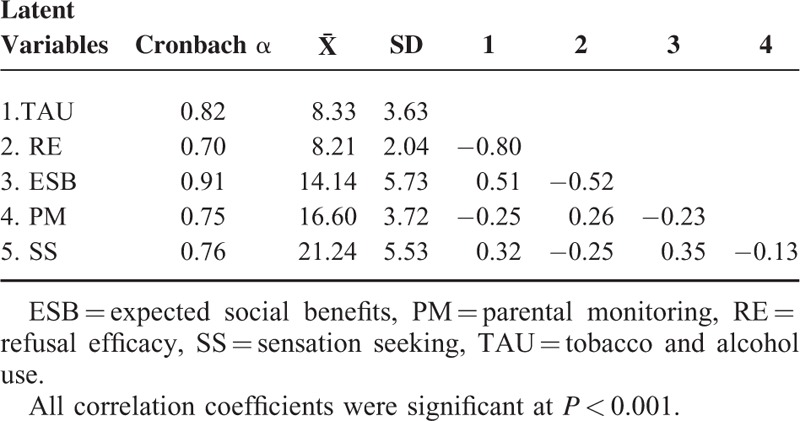
Chronbach Alphas, Descriptive Analyses, and Correlation Coefficients of Latent Variables

### Statistical Analysis

All data were entered into EpiData Version 3.1 (EpiData Association; Odense, Denmark) and a double-entry strategy was applied. Analogous to the Communities That Care Youth Survey,^30^ 3 strategies of data quality control were adopted for approximately 200 items, as well as some items involving illicit drug use in the survey. The 3 strategies are described below: first, false reporting was assessed via students’ responses to 2 items. One asked students whether they had honestly responded to all questions and another asked students the frequency of drinking water that might occur almost every day. These assessed whether the students had paid attention to the content of the questions. Second, logical conflicts were identified between the amount and the frequency of TAU (e.g., if a student's response to the amount of tobacco use was “I do not smoke,” but the response to the frequency of tobacco use was not “never”). Third, poor attitudes of students were evaluated. For instance, students answering questions following a set response rule, such as providing the same response to all items or not choosing any response to most of items in the survey, were eliminated. Any example of these inconsistencies was treated as unreliable data.

All reliable data were used for analyses. Descriptive analysis of demographic characteristics of the analyzed and deleted samples was conducted using SPSS version 20.0 (SPSS Inc, Chicago, IL). The SEM techniques were implemented with 2 steps using Mplus version 7.0 (Muthén & Muthén, Los Angeles, CA).^35^ The first step was to estimate the measurement model with confirmatory factor analysis (CFA). The second step was to estimate the structural model with sex, grade, residence, only child, and family structure as covariates. Age was not considered as a covariate given its close relationship with grade. Considering the simplicity of the model, each of the 2 similar descriptive items in ESB were parceled into 1 item.^36^ Meanwhile, the items belonging to the same subscale of BSSS-C were parceled into 1 item as well. The missing data for all variables were <1.0%, which was addressed with the Full Information Maximum Likelihood approach.^37^ Bootstrapping with 1000 iterations was used to estimate indirect effects and construct confidence intervals because it is a preferred strategy to test mediation and does not impose the assumption of normal distribution for samples.^38^

Four model fit indices were used to evaluate the fit of model. These indices were comparative fit index (CFI), Tucker-Lewis index (TLI), root mean square error approximation (RMSEA), and standardized root mean square residual (SRMR). The values of CFI and TLI >0.90, RMSEA ≤0.06, and SRMR ≤0.08 indicated adequate fit.^39^ The *P* < 0.05 was considered statistically significant.

## RESULTS

### Demographic Characteristics of Analyzed and Deleted Samples

A total of 1181 questionnaires were deleted based on the 3 strategies of data quality control described above (43.1%, 21.7%, and 35.2% for the first, second, and third strategy, respectively). As shown in Table 1, there were significant differences between analyzed and deleted samples in age, sex, residence, only child, and city. No significant differences were found in grade and family structure. For the analyzed samples, the proportions of students from Wuhan, Shenzhen, and Zhaoqing were 40.3%, 31.1%, and 28.6%, respectively. The age of the students ranged from 13 to 20 (M = 16.4, SD = 1.1). Approximately half (49.1%) of students were male. The proportions of students from the 10th and 11th grade were 52.0% and 48.0%. Urban students and only child accounted for 65.2% and 31.2%, respectively. In total, 77.8% of students lived with their parents most of time and 22.2% lived with only father or mother or other relatives.

**TABLE 1:**
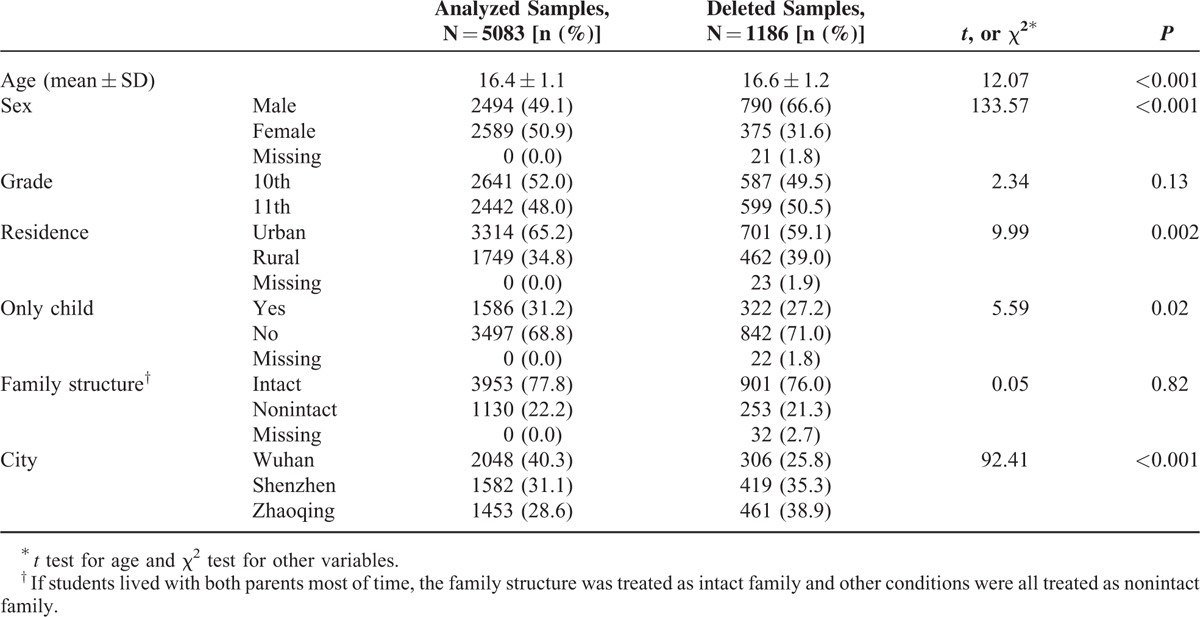
Comparison of Demographic Characteristics of Analyzed and Deleted Samples

### CFA

**FIGURE 3:**
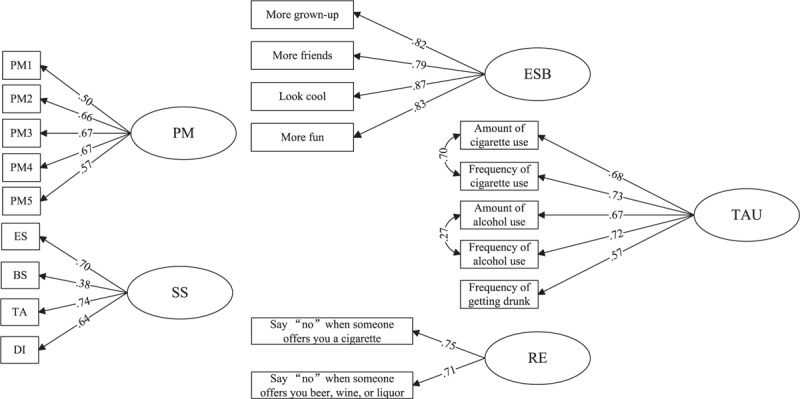
Confirmatory factor analysis of PM, SS, ESB, RE, and TAU among Chinese adolescents. Rectangles and ovals represented measured and latent variables, respectively. BS = boredom susceptibility, DI = disinhibition, ES = experience seeking, ESB = expected social benefits, PM = parental monitoring, RE = refusal efficacy, SS = sensation seeking, TA = thrill and adventure seeking, TAU = tobacco and alcohol use. See Supplementary Table 1, for indicators of latent variables. All factor loadings were standardized values and significant at *P* < 0.001. Standard errors, measurement errors, residual errors were not shown. Model fit indices: CFI = 0.947, TLI = 0.936, RMSEA = 0.045 (0.043–0.047), and SRMR = 0.036.

The fit indices of the initial measurement model estimate using CFA showed that model fit was not adequate: CFI = 0.896, TLI = 0.876, RMSEA = 0.062 (0.061–0.064), and SRMR = 0.053. Based on the theoretical knowledge and modification indices, the fit degree would be increased by allowing 2 sets of measurement errors to covary (i.e., the amount and frequency of tobacco use, as well as the amount and frequency of alcohol use). After these 2 changes, the fit indices were attained an adequate level: CFI = 0.947, TLI = 0.936, RMSEA = 0.045 (0.043–0.047), and SRMR = 0.036. As shown in Figure 3, all standardized factor loadings ranged from 0.38 to 0.87 and were significant at *P* < 0.001. Correlation coefficients among latent variables from CFA model are shown in Table 2. All coefficients revealed a moderate-to-strong correlation and the direction was as expected. In summary, the measurement model tested by CFA was properly specified in the present study, with adequate fit indices and acceptable factor loadings for each indicator variable.

### SEM

**FIGURE 4:**
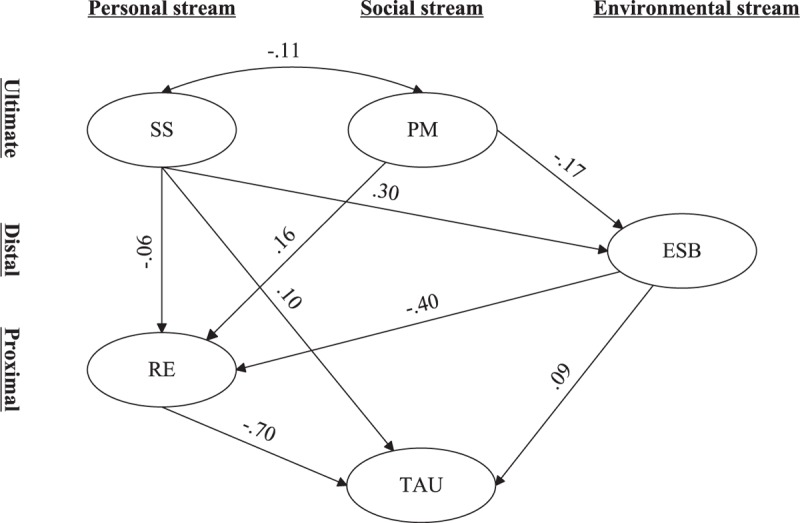
Final structural equation model consisted of PM, SS, ESB, RE, and TAU among Chinese adolescents. Covariates including sex, grade, residence, only child, and family structure were statistically controlled (paths and coefficients were not shown). Ovals represented latent variables. ESB = expected social benefits, PM = parental monitoring, RE = refusal efficacy, SS = sensation seeking, TAU = tobacco and alcohol use. All values were standardized coefficients and all coefficients were significant at *P* < 0.01. Standard errors, measurement models, and residual errors were not shown. Model fit indices: CFI = 0.942, TLI = 0.930, RMSEA = 0.045 (0.043–0.046), and SRMR = 0.032.

After estimation of the measurement model fit, the proposed structural model was subsequently tested with sex, grade, residence, only child, and family structure as covariates. The result of final structural model revealed an adequate model fit (see Figure 4). Compared with the original proposed model, one regression path depicting PM on TAU was omitted for its nonsignificant regression coefficient. The fit indices of the final model were acceptable: CFI = 0.942, TLI = 0.930, RMSEA = 0.045 (0.043–0.046), and SRMR = 0.032. The direction of all regression coefficients was as predicted. The model could explain 68.1% of the variance of TAU.

Standardized total, total indirect, specific indirect, and direct effects on TAU and mediator variables and 95% confidence intervals for each effect are presented in Table 3. The relationship between PM and TAU was completely mediated by ESB and RE (b = −0.18, *P* < 0.001). Meanwhile, SS was not only associated with TAU directly (b = 0.10, *P* < 0.001), but also indirectly through mediators ESB and RE (b = 0.15, *P* < 0.001). All mediation paths could explain 60.0% of the total effect. Furthermore, results showed that 75.7% of the total effect between ESB and TAU could be explained by the “ESB → RE → TAU” path (b = 0.28, *P* < 0.001). In addition, as shown in Figure 4, RE had the most influential effect on TAU (b = −0.70, *P* < 0.001).

**TABLE 3:**
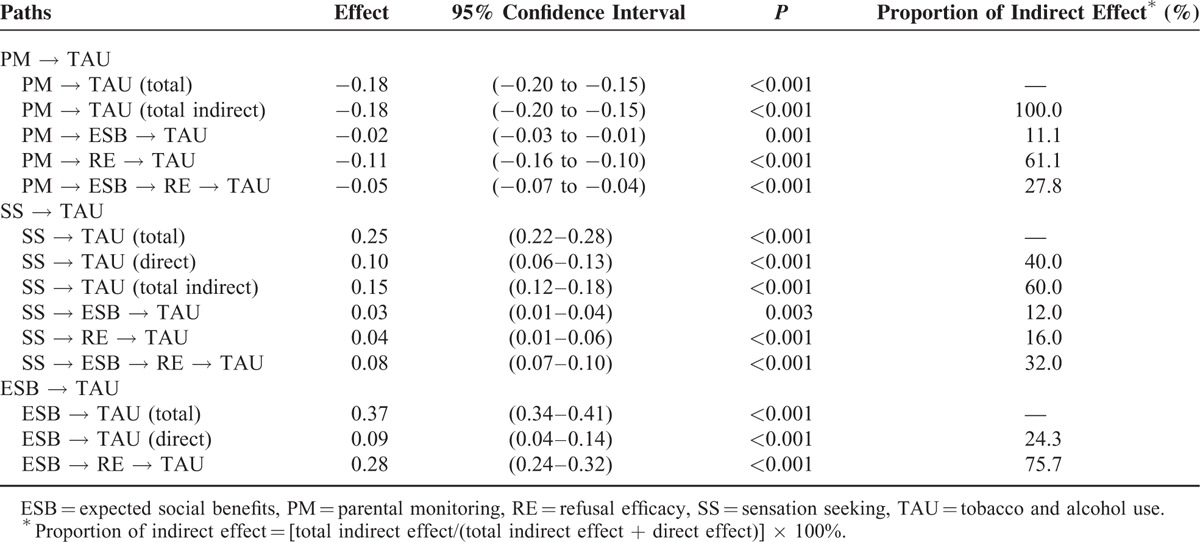
Standardized Total, Total Indirect, Specific Indirect, and Direct Effects on TAU and Mediator Variables and 95% Confidence Intervals for Each Effect

## DISCUSSION

The present study is a cross-sectional survey with strict quality control strategies among vocational high school students in China. To our knowledge, this is the first study to explore how PM, SS, ESB, and RE simultaneously influence TAU based on a theoretical framework (i.e., TTI). As expected, the SEM and mediation analyses results were consistent with the 3 primary hypotheses except for the direct effect of PM on TAU. ESB and RE mediated the relationships between PM, SS, and TAU. Meanwhile, RE mediated the relationship between ESB and TAU. Most variance of TAU (68.1%) could be explained by the model. These findings have important implications on the development of TAU prevention programs among Chinese adolescents.

Consistent with previous studies,^2,12,16,18,29^ the findings in the present study indicated that PM was a protective determinant to decrease adolescent TAU. This effect was completely mediated by ESB and RE. We speculate on some plausible reasons for these findings. First, parents with strictly monitored children have more chance to share the proper expectancies on TAU with their offspring. Second, greater monitoring on the children's whereabouts, friends, and activities by parents reduces the exposure to the scenes involving TAU-like recreational venues, with high risk for the formation of ESB. Third, low levels of PM would increase the opportunities for adolescents to associate with delinquent peers.^40^ They share similar beliefs, attitudes, values, and rationales for substance use, which could prompt them to form ESB toward substance use.^41^ These all in turn influence the use of tobacco and alcohol. In addition, high levels of PM help adolescents to establish safety boundaries, which are beneficial to them objectively understanding the risk of TAU.^42^ This would increase the adolescents’ confidence on resisting the tobacco and alcohol offers (i.e., RE) and then decrease the adolescent TAU.

To the contrary, SS was a risk determinant for adolescent TAU. This finding was in accordance with other studies.^10,13,14,32,43^ The effect was partially mediated by ESB and RE. These mediation effects might be explained for the following reasons. High SS adolescents are more sensitive to nicotine and alcohol, which creates a cognitive bias in learning positive effects of TAU.^44,45^ This could facilitate them forming stronger expectancies of social benefits toward TAU. Then, these positive expectancies are stored in the memory and further influence the use of tobacco and alcohol. Moreover, high sensation seekers could activate their memory of ESB more easily than low sensation seekers,^11,13^ which in turn increase the adolescent TAU. In addition, in order to satisfy the biological needs of stimulation, which could be provided by psychoactive drugs, high SS adolescents would be less likely to refuse the offers of tobacco and alcohol than low SS counterparts.^46,47^ This is also able to increase the adolescent TAU.

Unlike other research involving negative expectancies,^13,14,21,23^ we solely encompassed positive expectancies depicted as ESB in the present study. As is widely known, positive expectancy is a cognitive risk determinant for adolescent TAU. Our results suggested that ESB predominantly conveyed the risk for TAU through RE as a mediator, which supported previous findings.^23–25^ The higher ESB is placed on the tobacco and alcohol, the more difficult it is to refuse offers of engaging in TAU. This implies that ESB plays an important role on the decision of whether or not to use substances. In addition, our results also suggested RE was indeed a proximal predictor of TAU. As a cognitive protective determinant and the most specific factor to TAU, it mediated the effects of all other included determinants. This indicates the importance of RE to TAU prevention among Chinese adolescents.

Furthermore, as a macrolevel comprehensive theory, TTI has integrated a series of microlevel theories like the theory of planned behavior, social learning theory, health belief theory, etc.^9^ It has more strength for explaining the etiology of health-related behaviors. There is no existing theory covering all independent variables in the present study other than TTI. Another theory most closely related with this study might be the acquired preparedness model.^48,49^ In this model, positive expectancies mediate the relationship between personality traits (e.g., SS, impulsivity) and substance use. Hayaki et al have incorporated RE into the model, and the result demonstrated that RE was a mediator between impulsivity and marijuana problems.^21^ A similar result has been released in the current study. However, the variable PM is beyond the scope of this model.

It is noteworthy that there were demographic differences between analyzed and deleted samples. For mean age, residence, and only child, statistical significance has been found, but we consider it might be of no or little clinical significance because of the low magnitude of the differences (i.e., 0.2 years for mean age, 6.1% for residence, and 4.0% for only child). The city difference might be explained by different investigators. Although there was similar training for all investigators (student investigators for Zhaoqing city and adult investigators for the other 2 cities), a difference still existed. This might represent a perception that the adult investigators represent authority figures and so produce an altered response rate. Finally, sex differences might contribute to personality characteristics. Generally, Chinese male adolescents are regarded as more extroverted and stubborn, whereas female adolescents are more easily persuaded.^50^ These could lead to enhanced cooperation by female students than male.

Important limitations to the current study are as follows. First, it is difficult to determine causality from the cross-sectional design. Longitudinal studies are needed to test the model from the present study. Second, analytic data were collected from students with self-report questionnaires. Although the principle of confidentiality was stressed, bias and underestimation might still exist because some students were unwilling to report real information.^51^ Third, though all scales in the present study reached an adequate level of reliability, the Cronbach alphas of PM, SS, and RE were relatively low (Cronbach α = 0.75, 0.76, and 0.70, respectively). In order to obtain a good prediction of these variables to TAU, higher reliability scales might be adopted in future studies. Fourth, the findings in this study should be interpreted with caution for 2 reasons. A small number of out-of-school adolescents might be a high-risk group more likely to use/abuse alcohol and tobacco; however, they were obviously not included in the current study. On the contrary, there were approximately 20% of selected students’ data deleted following our quality control strategies and the demographic characteristics between 2 samples were not completely balanced. More strategies should be taken to enhance the degree of student cooperation in the future. In the present study, all these demographic characteristics have been considered as covariates.

Despite these limitations, our findings still guide the direction for designing TAU prevention programs among Chinese adolescents. For parents, training for monitoring skills should be especially stressed in the programs. Moreover, adolescents with high SS characteristic or perceiving low levels of PM should be targeted by the programs. Because ESB and RE are 2 important mediators, programs should also strongly emphasize to adjust these 2 cognitive components (i.e., correcting misperceptions of ESB and increasing RE) among high risk adolescents. Some good experiences from previous programs are worth noting. A prevention program focusing on strengthening PM and participation in organized sports has been reported to be effective in protecting adolescents against substance use in Iceland.^52^ Other studies have identified the effectiveness of brief personality-targeted (including SS) interventions in preventing adolescent substance misuse.^53^ Furthermore, a school-based project named Life Skills Training (LST) has been established to be effective in adolescent tobacco, alcohol, and other drug use prevention.^54^ Two important goals of LST are to correct misperceptions about substance use and promote RE.

## CONCLUSION

In summary, this is a cross-sectional study following the theoretical framework of TTI. The results verify and expand the mechanisms how PM, SS, ESB, and RE influence TAU among Chinese adolescents. The findings indicate that the underlying mechanisms linking PM and SS to TAU among Chinese adolescents can be explained by ESB and RE. These 4 precursory determinants are important points for the development of adolescent TAU prevention programs, which has been rarely addressed in China. This therefore represents an important area for intervention development, and the present study provides important information to inform the creation of such approaches.

## Supplementary Material

Supplemental Digital Content
