# Supplementary material for: Influence of Parental Monitoring, Sensation Seeking, Expected Social Benefits, and Refusal Efficacy on Tobacco and Alcohol Use in Chinese Adolescents
Source: Medicine (Baltimore). 2016 Mar 18;95(11):e2814. doi: 10.1097/MD.0000000000002814 (PMC4839879; doi:10.1097/MD.0000000000002814)
Supplement: Supplemental Digital Content [file medi-95-e2814-s001.pdf]

Influence of parental monitoring, sensation seeking, expected social benefits, and refusal efficacy on tobacco and alcohol use in Chinese adolescents

Supplementary Table 1: Latent variables and items for Structural Equation Modeling analyses

Jincong Yu

Department of Epidemiology and Biostatistics, School of Public Health,

Tongji Medical College, Huazhong University of Science and Technology,

No.13 Hangkong Road, Qiaokou District, Wuhan, China

Supplementary Table 1 Latent variables and items for Structural Equation Modeling analyses

Latent variable	Item
Tobacco and alcohol use (TAU)	How often (if ever) do you smoke a cigarette?
	How often (if ever) do you consume alcoholic beverages (i.e. beer, wine, hard liquor)?
	How often (if ever) do you get drunk?
	How many cigarettes do you have on a typical day when you smoke?
	How many drinks do you have on per drinking occasion?
Refusal efficacy (RE)	How likely would you be to say 'no' when someone offers you a cigarette?
	How likely would you be to say 'no' when someone offers you beer, wine, or liquor?
Expected social benefits (ESB)	Adolescents who smoke cigarette are more grown-up.
	Adolescents who drink alcohol are more grown-up.
	Adolescents who smoke cigarette have more friends.
	Adolescents who drink alcohol have more friends.
	Smoking cigarette makes you look cool.
	Drinking alcohol makes you look cool.
	Smoking cigarette lets you have more fun.
	Drinking alcohol lets you have more fun.
Parental monitoring (PM)	The rules in my family are clear.
	When I am not at home, one of my parents knows where I am and whom I am with.
	My parents ask if I've gotten my homework done.
	My parents would know if I did not come home on time.
	If I skipped school, I would be caught by my parents.
Sensation Seeking (SS)	I'm interested in almost everything that is new.
	I always like to do things that no one else has done before.
	Take adventures always makes me happy.
	I would love to socialize with adventurous people.
	I get restless if I do the same thing for a long time.
	I will feel very uncomfortable if I stay in the same place for too long.
	I would do anything as long as it exciting and stimulating.
	I would love to have new and exciting experiences, even if they are against rules.
